# Unresponsive wakefulness syndrome: a new name for the vegetative state or apallic syndrome

**DOI:** 10.1186/1741-7015-8-68

**Published:** 2010-11-01

**Authors:** Steven Laureys, Gastone G Celesia, Francois Cohadon, Jan Lavrijsen, José León-Carrión, Walter G Sannita, Leon Sazbon, Erich Schmutzhard, Klaus R von Wild, Adam Zeman, Giuliano Dolce

**Affiliations:** 1 Coma Science Group, Dept of Neurology and Cyclotron Research Centre, University Hospital and University of Liège, 4000 Liège, and Belgian National Science Funds, Belgium; 2 Dept of Neurology, Loyola University of Chicago, Stritch School of Medicine, 2160 S. First Avenue, Maywood, IL 60153, USA; 3 Neurosurgical University Hospital, Pellegrin Tripode, Bordeaux, France; 4 Dept of Primary and Community Care, Radboud University Nijmegen Medical Centre Nijmegen, The Netherlands; 5 Dept of Experimental Psychology, University of Seville, Spain; Center for Brain Injury Rehabilitation, Torneo 23, Seville, Spain; 6 Department of Neuroscience, Ophthalmology and Genetics, University of Genova, Genova, Italy; 7 Department of Psychiatry and Behavioral Science, State University of New York, Stony Brook, NY, USA; 8 Tel Aviv University, Sackler Medical School, or Former Director of ICU for Vegetative Patients at Loewenstein Rehabilitation Hospital, Israel; 9 Dept of Neurology, University Hospital Innsbruck, Austria; 10 Medical Faculty, Westphälische Wilhelms-University, Münster, Germany; 11 Department of Rheumatology and Rehabilitation, Al-Azhar University, Cairo, Egypt; 12 Cognitive and Behavioral Neurology, Peninsula Medical School, Exeter, UK; 13 Research on Advanced Neuro-rehabilitation, S. Anna Institute, Via Siris - IT-88900 Crotone, Italy

## Abstract

**Background:**

Some patients *awaken *from coma (that is, open the eyes) but remain unresponsive (that is, only showing reflex movements without response to command). This syndrome has been coined *vegetative state*. We here present a new name for this challenging neurological condition: *unresponsive wakefulness syndrome *(abbreviated UWS).

**Discussion:**

Many clinicians feel uncomfortable when referring to patients as *vegetative*. Indeed, to most of the lay public and media *vegetative state *has a pejorative connotation and seems inappropriately to refer to these patients as being *vegetable-like*. Some political and religious groups have hence felt the need to emphasize these vulnerable patients' rights as human beings. Moreover, since its first description over 35 years ago, an increasing number of functional neuroimaging and cognitive evoked potential studies have shown that physicians should be cautious to make strong claims about awareness in some patients without behavioral responses to command. Given these concerns regarding the negative associations intrinsic to the term *vegetative state *as well as the diagnostic errors and their potential effect on the treatment and care for these patients (who sometimes never recover behavioral signs of consciousness but often recover to what was recently coined a *minimally conscious state*) we here propose to replace the name.

**Conclusion:**

Since after 35 years the medical community has been unsuccessful in changing the pejorative image associated with the words *vegetative state*, we think it would be better to change the term itself. We here offer physicians the possibility to refer to this condition as *unresponsive wakefulness syndrome *or UWS. As this neutral descriptive term indicates, it refers to patients showing a number of clinical signs (hence *syndrome*) of unresponsiveness (that is, without response to commands) in the presence of wakefulness (that is, eye opening).

## Background

We here present a new name (*unresponsive wakefulness syndrome *or UWS) for an over 35-year-old syndrome with an unintended albeit persistent negative connotation: the vegetative state. The widespread use of intensive care medicine and artificial ventilation to sustain respiration and circulation has increased survival from coma. It has also led to an increasing number of patients who have *awakened *from coma (that is, showed eye opening, incompatible with the diagnosis of coma) yet remain unresponsive (that is, only showed reflex movements as is also the case for coma) [1]. In Europe, this clinical syndrome was initially termed *apallic syndrome *[2] and *coma vigil *[3] but it is currently known in the medical community as *persistent vegetative state *(PVS), a term first coined by Jennet and Plum in 1972 in their milestone *Lancet *paper [4]. The name *vegetative state *was chosen to refer to the preserved vegetative nervous functioning, meaning these patients have (variably) preserved sleep-wake cycles, respiration, digestion or thermoregulation. The term *persistent *was added to denote that the condition remained for at least one month after insult. In 1994, the Multi-Society Task Force on PVS defined the temporal criteria for irreversibility (that is, more than one year for traumatic and three months for non-traumatic (anoxic) etiology) and introduced the notion of *permanent vegetative state *[5]. It is to these latter cases that ethical and legal end-of-life issues, of withholding and withdrawal of life sustaining treatment (that is, artificial hydration and nutrition), are related [6,7].

Over the last three decades, a growing number of physicians and healthcare workers have felt uncomfortable when referring to patients as *vegetative *[8-10], resulting in a number of papers reiterating the intellectual justification of the origins and choice of the term [11]. The conception of a *vegetative nervous system *goes back to 1800 when Bichat divided the nervous system into animalic and vegetative [12]. The former linked the person to her or his environment and was expressed by the muscles of voluntary locomotion and the organs of external senses. The latter identified the nutritional functions of the body. According to the Oxford English dictionary, '*to vegetate' *is to 'live a merely physical life devoid of intellectual activity or social intercourse' and '*vegetative' *describes 'an organic body capable of growth and development but devoid of sensation and thought'. To part, if not most, of the lay public and media, however, it has a rather pejorative undertone and seems (incorrectly) to refer to patients as being *vegetable-like *(for example, an internet search with the terms *vegetative state *and *vegetable *returned 26,700 hits, *état végétatif *and *plante *19,600; *stato vegetativo *and *vegetale *49,100 (Google search performed 8 April 2010). Many authors and social, political and religious groups have hence felt the need to emphasize these patients' clearly evident rights to be fully regarded as human beings [13,14].

In addition to this malaise regarding the chosen term and its unintended denigrating connotation, some feel that referring to these patients as being in a *state *may (incorrectly) denote chronicity. Despite the fact that the clinical criteria of the vegetative state do not imply a temporal dimension, referring only to a clinical tableau reflecting *wakeful unawareness *[4], for many physicians and healthcare workers it has the negative connotation of a being a longstanding and nearly irreversible condition. The introduction of the term *persistent vegetative state *(too often confounded with *permanent vegetative state *with which it unfortunately shares the same abbreviation PVS), may have contributed to this [15]. In contrast to coma (which is an acute and transitory condition, lasting no more than days or weeks), a *vegetative state *may become chronic (lasting for decades) or may remain a transitory condition on the way to further recovery [16]. This recently led the *Aspen Neurobehavioral Conference Workgroup *to characterize a new clinical entity coined the 'minimally conscious state' (MCS), describing patients who have recovered from a *vegetative state *(meaning they show more than reflex motor behavior but fail to show functional communication or object use) [17]. Despite clear evidence that *vegetative *patients are not uniformly hopeless [18,19], once stamped with the diagnosis *VS*, clinical practice shows it often is difficult to change the label, and the first signs of recovery of consciousness are too often missed. Previous studies by Childs *et al*. in Texas [20] and Andrews *et al*. in London [21] have estimated misdiagnosis of chronic patients referred to rehabilitation centers to be at around 40%. It has been argued that these older studies, performed prior to the publication of the Multi-Society Task Force on PVS criteria [5] of VS, and long before the criteria of the MCS [17], were overly pessimistic. A very recent study, however, confirmed this unacceptably high rate of diagnostic error [22]. A number of highly publicised patients also illustrate this point. Julia Tavalaro survived a brain trauma and was transferred to a tertiary care centre where she was called "*the vegetable*" for over six years, although she was conscious and sensate. She later wrote her memoirs in *Look Up for Yes *[23]. Terry Wallis, who was considered to be in a VS, made the headlines when he started to speak 19 years after his car accident. Careful analysis of his medical records quickly showed he actually recovered to a MCS within the first year after his brain trauma [24]. Finally, since the term VS was coined in 1972, an increasing number of functional neuroimaging and event related potential (ERP) studies have shown that physicians should be very careful about making strong claims about patients' awareness [25-31]. This situation is further complicated when patients with such disorders of consciousness have underlying deficits in the domain of verbal or non-verbal communication functions, such as aphasia, agnosia or apraxia [32,33].

## Discussion

Given these concerns regarding the negative connotation inherent in the term *vegetative state *and its possible effect on vulnerable patients awakening from coma, who sometimes never recover any voluntary responsiveness but may (probably more often than initially believed) recover minimal signs of consciousness, we here propose to change the label *vegetative state*, thus hoping to make it easier to change their management and standards of care. The European Task Force on Disorders of Consciousness has passed a proposal to change the name to *unresponsive wakefulness syndrome *or UWS. If after 35 years the medical community has been unsuccessful in changing the pejorative image associated with the words vegetative *state*, we propose that it might be better to change the term itself. From now on, physicians can choose this neutral descriptive term to refer to patients who, as the name indicates, show a number of clinical signs (hence the use of *syndrome*) of unresponsiveness (meaning they fail to show non-reflex behavior or command following) in the presence of wakefulness (meaning they open their eyes spontaneously or upon stimulation). Given the above mentioned difficulty in making strong general claims about awareness in severely brain damaged patients, we have chosen here to use the clinically descriptive term *unresponsive *rather than the misleading *unaware*. After discussion, other (existing) alternatives [34] were rejected. *Coma vigil *was discarded because the term is a contradiction in terminis, given that coma patients by definition never open their eyes. *Apallic syndrome *was also rejected, as recent evidence has shown that these patients are not a-pallic (meaning without a cortex or pallium) [35], but classically show preserved albeit disconnected islands of residual (merely primary) cortical functioning [36].

Next, we stress the need for prospective studies on prognosis [18,37,38] and treatment [39,40] in large, well-described cohorts of patients with disorders of consciousness, permitting evidence-based decision-making while respecting individual divergence in the challenging issues related to end-of-life decisions [6,7]. Such studies will need standardized behavioral assessment and outcome scales [41]. The worldwide acceptance of the Glasgow Coma Scale (GCS) [42] has standardized patient assessment in the ICU and allowed proper research to be carried out in the field of coma. However, the GCS was not intended to be used on patients with post-comatose disorders of consciousness, such as UWS and MCS. Other standardized scales will need to be employed in these cases [43,44]. We also need reliable objective para-clinical markers confirming our clinical signs of motor unresponsiveness and behavior indicative of the absence of awareness of environment and self [45]. Studies assessing the efficacy of treatment of patients with disorders of consciousness should be separated into symptomatic and curative and should take into account not only patient age, etiology and time since insult, but also the need to clearly separate UWS from MCS [46].

## Conclusion

In conclusion, our proposal offers the medical community the possibility to adopt a neutral and descriptive name, *unresponsive wakefulness syndrome*, as an alternative to *vegetative state *(or *apallic syndrome*) which we view as outdated. We feel this is a real necessity, given that the term PVS continues to have strong negative connotations after over 35 years of use, while inadvertently risking comparisons between patients and vegetables and implying persistency from the moment of diagnosis. It should be stressed that UWS is a clinical syndrome describing patients who fail to show voluntary motor responsiveness in the presence of eyes-open wakefulness which can be either transitory on the way to recovery from (minimal) consciousness or irreversible.

## Abbreviations

ERP: event related potential; GCS: Glasgow Coma Scale; MCS: minimally conscious state; PVS: persistent vegetative state; UWS: unresponsive wakefulness syndrome; VS: vegetative state

## Competing interests

The authors declare that they have no competing interests.

## Authors' contributions

All authors contributed to the content of this manuscript and have read and approved the final draft.

## Pre-publication history

The pre-publication history for this paper can be accessed here:

http://www.biomedcentral.com/1741-7015/8/68/prepub
